# Refractory Acute Respiratory Distress Syndrome Secondary to COVID-19 Successfully Extubated to Average Volume-assured Pressure Support Non-invasive Ventilator

**DOI:** 10.7759/cureus.7849

**Published:** 2020-04-27

**Authors:** Abhinav Mittal, Michael Forte, Rachel Leonard, Rahul Sangani, Sunil Sharma

**Affiliations:** 1 Pulmonary, Critical Care & Sleep Medicine, West Virginia University, Morgantown, USA; 2 Internal Medicine, Section of Pulmonary, Critical Care & Sleep Medicine, West Virginia University, Morgantown, USA

**Keywords:** covid 19, non-invasive mechanical ventilation, avaps, worsening obstructive sleep apnea (osa), critical illness myopathy, sars-cov-2, pulmonary hypertension

## Abstract

Coronavirus disease 2019 (COVID-19) is a respiratory illness caused by the highly infectious novel SARS-CoV-2 coronavirus spread by droplet transmission. Consequently, the use of respiratory devices that may potentially promote aerosolization like non-invasive positive pressure ventilation (NIPPV) for diseases such as obstructive sleep apnea (OSA), advanced chronic obstructive lung disease, pulmonary hypertension (PH), and neuromuscular respiratory disease has been called into question. We present a case of a patient with history of OSA and PH convalescing from refractory acute respiratory distress syndrome (ARDS) secondary to COVID-19 who was successfully extubated to average volume-assured pressure support (AVAPS).

A 74-year-old male with medical history notable for OSA on NIPPV, PH, and hypertension presented with respiratory failure secondary to COVID-19 confirmed on polymerase chain reaction (PCR) test. His respiratory status worsened leading to ARDS requiring intubation. He was initially extubated to high flow nasal cannula (HFNC) due to hospital policy to avoid NIPPV due to concerns of viral dissemination. He did not tolerate HFNC and required re-intubation for prolonged period. He was then medically optimized for a second attempt and extubated two days later to AVAPS with an anti-viral filter and negative pressure room with a goal of optimizing his critical illness myopathy and pre-existing OSA and PH. He tolerated extubation well, and over the next five days was weaned from alternating AVAPS/HFNC to eventually requiring two liters nasal cannula in the day and AVAPS mode at night.

This case highlights a potential therapeutic option for patients with severe respiratory failure secondary to COVID-19. This patient’s pre-existing comorbidities of OSA and PH markedly increased his risk for extubation failure on HFNC. The use of AVAPS after his second extubation attempt helped ensure ventilation and oxygenation non-invasively. COVID-19 can lead to prolonged dependence on mechanical ventilation. This pandemic has the potential to create medical resource scarcities, especially in rural areas where ventilators and trained personnel are already in short supply. By using AVAPS mode, this patient was able to rehabilitate his myopathy and participate in intermittent weaning of HFNC to ultimately simple nasal cannula.

AVAPS is useful tool to facilitate extubation, as it allows non-invasive support of respiratory dynamics, particularly in those with co-morbidities such as OSA and PH. Further, larger scale studies are needed to determine its exact role during the COVID-19 pandemic.

## Introduction

The management of coronavirus disease 2019 (COVID-19) patients with hypoxemic respiratory failure is challenging due to high infectious potential of novel SARS-CoV-2, presentation heterogeneity, severity of disease manifestation, finite supply of ventilators, and limited evidence-based practices [1,2]. Given uncertainties regarding modes of transmission and risk of aerosolization of this novel virus, the use of non-invasive positive pressure ventilation (NIPPV) has been called into question [3]. However, there is currently little guidance on how clinicians should manage patients previously reliant on NIPPV for diseases such as obstructive sleep apnea (OSA), advanced chronic obstructive lung disease, pulmonary hypertension (PH), and neuromuscular respiratory disease. Not only are patients with these co-morbidities more likely to suffer more serious manifestations of COVID-19, but prolonged ventilation predisposes patients to neuromuscular weakness. Strategic use of average volume-assured pressure support (AVAPS) mode of ventilation as a bridge in recovering COVID-19 has the potential to facilitate earlier extubation, conserve traditional ventilators, and prevent re-intubation/tracheostomies. We present a case of a patient with history of OSA and PH convalescing from refractory acute respiratory distress syndrome (ARDS) secondary to COVID-19 who was successfully extubated to AVAPS.

## Case presentation

**Figure 1:**
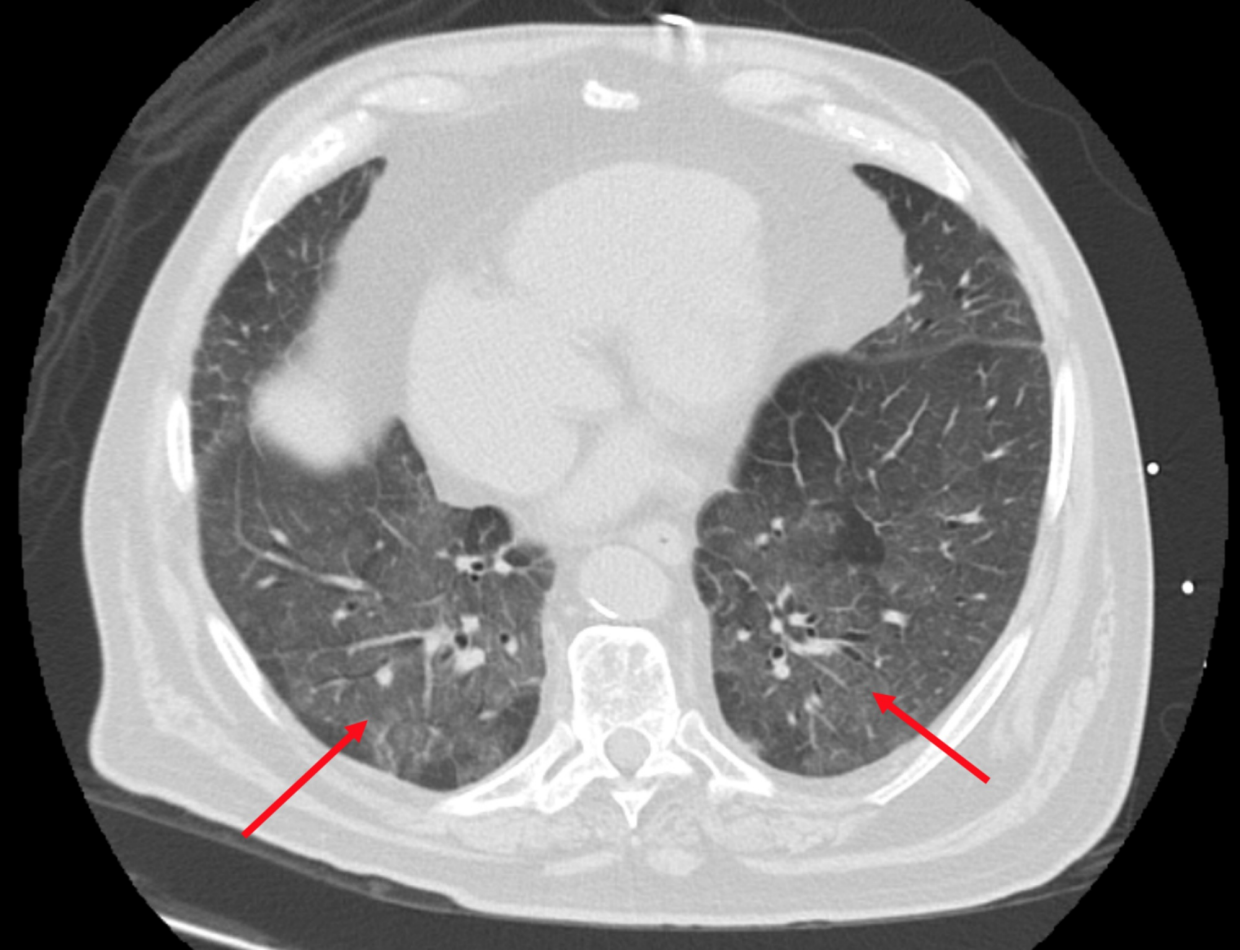
Computed tomography chest, axial image showing bilateral ground glass opacities (red arrows) with peripheral and basilar predominance typical of COVID-19.

A 74-year-old male with medical history notable for OSA on NIPPV, PH, and hypertension initially presented to an outside hospital due to hypoxia and dyspnea. A week prior, he was in great functional health and, in fact, was returning from a skiing trip requiring travel through two major US airports. Initial workup demonstrated bilateral infiltrates on chest imaging (Figure 1) and COVID-19 polymerase chain reaction (PCR) test was positive.

His respiratory status worsened leading to moderate ARDS with PaO2/FiO2 (arterial oxygen partial pressure divided by fraction of inspired oxygen) ratio of 109 prompting intubation, paralysis, and transfer to our tertiary care facility. His intensive care unit (ICU) stay was complicated by ventilator-associated pneumonia, non-ST elevation myocardial infarction (NSTEMI), delirium, and myopathy of critical illness. Transthoracic echocardiography showed preserved left ventricular ejection fraction after NSTEMI and he was medically optimized over the next week. Standard ARDS management helped to slowly improve his respiratory status, and on hospital day 6 (day 9 of intubation), he was extubated to high flow nasal cannula (HFNC) because hospital policy recommended avoidance of NIPPV. Unfortunately, he failed the trial and required re-intubation. He was again medically optimized and two days later was on minimal ventilator settings and favorable weaning parameters. However, this time given our concerns for his critical illness acquired myopathy and to better address his pre-existing OSA and PH, we gained institutional clearance to proceed with trial of extubation to AVAPS with an anti-viral filter and negative pressure room on intubation day 11. He tolerated extubation well and over the next five days was weaned from AVAPS/HFNC alternating to eventually requiring two liters nasal cannula in the day and AVAPS mode at night.

## Discussion

This case highlights a potential therapeutic option for those patients with respiratory failure from COVID-19. This patient’s pre-existing co-morbidities of OSA and PH markedly increased his risk for extubation failure on the first attempt. While HFNC can potentially provide some positive end expiratory pressure, it is certainly inferior to NIPPV [4]. The use of AVAPS allowed us to ensure ventilation and oxygenation in a similar manner to mechanical ventilation non-invasively. Furthermore, by using AVAPS mode, the patient was able to rehabilitate his myopathy and participate in intermittent weaning of HFNC to ultimately simple nasal cannula. We continued AVAPS therapy nightly for treatment of his OSA and PH. We believe a similar scheme of ventilator weaning would be helpful in other such patients.

COVID-19 is a respiratory illness leading to hypoxic respiratory failure with rates of about 19% including need for NIPPV in 4-13% and intubation in 2-12% [5]. Patients with severe COVID-19 infections often have prolonged dependence on mechanical ventilation. Prolonged duration of mechanical ventilation predisposes patients to critical illness neuropathy/myopathy sometimes referred to as "ICU acquired weakness". ICU-acquired weakness increases risk of in-ICU, in-hospital, and long-term mortality, along with duration of mechanical ventilation, and length of stay. It augments healthcare-related costs, increases likelihood of prolonged care in rehabilitation centers, and reduces physical function and quality of life in the long term [6]. In such a circumstance, the need to consider long-term tracheostomy may be superseded by the risk of further aerosolization involved in directly manipulating the airway of a COVID-19 positive patient. Our use of non-invasive ventilation with AVAPS mode not only helped optimize this patient’s co-morbidities, but also ultimately avoided tracheostomy. We believe the AVAPS mode was able to provide him sufficient ventilatory support which kept him extubated. Liberation from mechanical ventilation allowed him to better participate in rehabilitation for a better prognosis. This mode of ventilation could potentially shorten intubation times and reduce risk of neuromuscular weakness.

AVAPS is a relatively new mode of ventilation. AVAPS uses an internal algorithm to make changes in the pressure support supplied to achieve the target volume, but these changes are small and occur over minutes (typically 1-2.5 cm water per minute). That is why it is not a good mode in acute setting with rapid respiratory rate - but as noted in our case - worked well in a recovering patient requiring a slow wean. The perceived advantage of AVAPS over bilevel positive airway pressure (BIPAP) includes maintaining volumes despite altered patient effort based on sleep stage or altered lung mechanics. A fixed pressure support setting is unlikely to compensate for these changes in resistance due to changing lung compliance, body position, and sleep. In a randomized trial, AVAPS delivered a lower mean PS for oxygenation and transcutaneous PaCO2 levels and promoted better adherence than BiPAP [7]. AVAPS mode is typically used in patients with neuromuscular disorders, OHS and chronic obstructive pulmonary disease (COPD).

Given the potential impact of this pandemic to exhaust medical resources like ventilators, discussions of possible salvage support strategies like synchronous mechanical ventilation of multiple patients with a single ventilator are gaining attention [8]. Such pandemic-related scarcities have serious ramifications in rural areas where ventilators and trained personnel are already in short supply. Utilization of home ventilators with AVAPS mode as a means of stepping down ventilator support could be one option to allocate the more powerful/sophisticated ventilators to the sickest patients. Given this successful extubation after nearly two weeks on the ventilator, this experience also lends credence to the possibility of using AVAPS and other NIPPV devices to support patients on minimal ventilator settings who need more time convalescing from their acute illness [9].

## Conclusions

This case highlights the potential utility of AVAPS as useful tool to facilitate extubation, with its ability to manage complex lung dynamics including muscular weakness inherent to prolonged ventilation. Using AVAPS as a bridge facilitated successful extubation and avoided tracheostomy. This could further help liberate ventilators for more advanced ARDS patients. Further, larger scale studies are needed to determine its exact role during the COVID-19 pandemic.
